# Low vitamin D levels and the long‐term functional outcome of stroke up to 5 years

**DOI:** 10.1002/brb3.2244

**Published:** 2021-09-02

**Authors:** Ya‐Ying Zeng, Cheng‐Xiang Yuan, Meng‐Xuan Wu, Lin Cheng, Sheng‐Nan Zhou, Ping‐lang Hu, Kai‐Li Fan, Wen‐Jie Tang, Jin‐Cai He

**Affiliations:** ^1^ Department of Neurology The First Affiliated Hospital of Wenzhou Medical University Wenzhou China; ^2^ School of Mental Health Wenzhou Medical University Wenzhou China; ^3^ First School of Clinical Medicine Wenzhou Medical University Wenzhou China

**Keywords:** functional outcome, ischemic stroke, prognosis, stroke, vitamin D

## Abstract

**Background and purpose:**

Previous studies have established that vitamin D was associated with stroke. The purpose of this study was to investigate the relationship between vitamin D and 5‐year outcome of patients with stroke including acute ischemic stroke (AIS) and intracranial hemorrhage (ICH) stroke.

**Methods:**

Serum 25‐hydroxyvitamin D levels were prospectively analyzed in patients admitted to the First Affiliated Hospital of Wenzhou Medical University from 2013 to 2015. Modified Rankin scale (mRS) was used to evaluate their 5‐year functional outcome, and univariate and multivariate logistic regressions were applied to evaluate the effects of vitamin D on stroke outcome.

**Results:**

In total, 668 patients diagnosed with stroke were recruited, and 420 completed the 5‐year follow‐up. Ninety‐five patients experienced poor outcome in the 5 years since stroke onset. Vitamin D levels in patients with poor outcome showed significant differences compared to good outcome patients (*p *< .001). In multivariable logistic regression analysis, after adjusting the potential confounders, the 5‐year functional outcome was significantly associated with vitamin D levels. Stroke patients with vitamin D levels less than 38.4 nmol/L had a higher risk for poor outcome compared with those with vitamin D level over 71.4 nmol/L at 5‐year (odds ratio [OR] = 3.66, 95% confidence interval [CI] = 1.42–9.45, *p* = .007), which was consistent with AIS patients (OR = 6.36, 95% CI = 1.89–21.44, *p* = .003).

**Conclusion:**

Vitamin D level less than 38.4 nmol/L at admission is a potential risk biomarker for poor functional outcome at 5‐year prognosis in AIS patients, which might provide new ideas for the prognostic assessment of stroke.

## 1 INTRODUCTION

Stroke is generally considered as the second leading cause of death and a first leading cause of adult disability worldwide. Around 2.5 million people are suffering from stroke each year in China, laying heavy burden on the families, communities, and healthcare systems (Holick et al., 2011; Wang et al., 2014). Survivors often need long‐term rehabilitation, but most stroke survivors still lead to poor outcomes. Therefore, early identification of risk factors for poor outcome and improving the functional prognosis are essential.

The recent research on stroke outcomes suggests that several factors related to the outcome of stroke had been identified, including age, sex, stroke severity, etiology, and cardiovascular risk factors (Robinson et al., 2019; Siegler et al., 2013; Torres‐Aguila et al., 2019; Werner et al., 2005). Other studies also suggested different stroke outcomes varied depending on the age stratification (Buell & Dawson‐Hughes, 2008; Razmara et al., 2017; Ueno et al., 2018). However, there is limited research on the relationship between vitamin D and long‐term functional outcomes of stroke.

Cholecalciferol (Vitamin D3) is the main form of vitamin D in nature, which is formed by the animals and human skin as a reaction to sunlight and could be obtained through diet (Krishna, 2019). Given that the cutaneous production of vitamin D decreases with age and the older are less exposed to sun, vitamin D deficiency becomes more common in stroke patients (Krishna, 2019; MacLaughlin & Holick, 1985). Previous studies have shown that lower 25‐hydroxyvitamin D (25(OH)D) levels were associated with poorer functional outcomes and all‐cause mortality in stroke patients (Chen et al., 2018; Daubail et al., 2013; Park et al., 2015; Tu et al., 2014; Wei & Kuang, 2018). Recent evidence has also suggested the negative results of low 25(OH)D levels on the survival of patients with different diseases, including myocardial infarction (Correia et al., 2013), heart failure (Liu et al., 2011), and chronic kidney disease (Pilz, Iodice, et al., 2011). Most studies on vitamin D and its outcomes have only focused on the short‐term prognosis after stroke. Large‐scale studies are in high demand concerning the functional prognostic value of stroke patients with serum 25(OH)D after 5 years.

To date, the relationship between vitamin D and functional outcome 5 years after stroke has not been illustrated, and it still remains unclear whether vitamin D has a strong effect on long‐term prognosis. As a result, this study was designed to investigate the association between vitamin D stratification and outcome among acute ischemic stroke (AIS) and intracranial hemorrhage (ICH) stroke after 5 years.

## 2 MATERIALS AND METHODS

### 2.1 Study design

From October 2013 to February 2015, consecutive stroke patients without any pre‐morbid handicap were admitted to the First Affiliated Hospital of Wenzhou Medical University. All participants agreed with the written informed consent. This study was approved by the Ethics Committee of the First Affiliated Hospital of Wenzhou Medical University (number: 2019−042) and conformed to the Helsinki Declaration.

Criteria of patient enrollment were as follows: (1) age between 18 and 80 years; (2) acute stroke occurred within 7 days before admission; and (3) diagnosed with computerized tomography (CT) or magnetic resonance imaging (MRI) at the time of admission.

The exclusion criteria were as follows: (1) autoimmune diseases; (2) patients with any central nervous system disease including dementia or severe cognitive impairment, and Parkinson's disease; (3) pre‐stroke major depression or other psychiatric disorders; (4) severe hepatic or renal diseases; (5) transient ischemic attack; (6) malignant tumor; (7) patients with osteoporosis or taking vitamin D supplementation; and (8) patients with stroke were treated with thrombolysis or mechanical thrombectomy.

**FIGURE 1:**
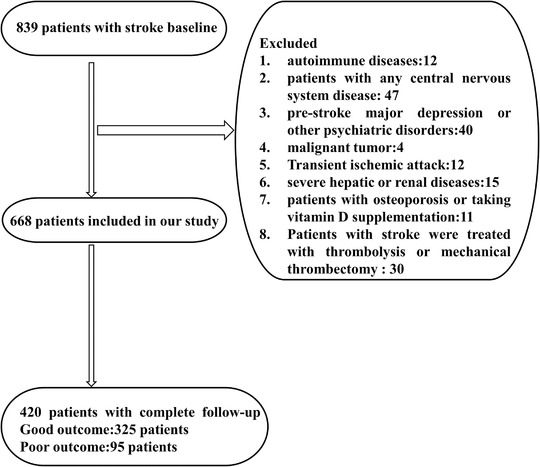
Study flow diagram

A total of 668 stroke patients were enrolled in the study at admission and 420 of that completed a 5‐year follow‐up. Patients were assessed at 1 month, 3 months, and 5 years following stroke until August 2019. The mean length of the following time since index stroke was 5 years (range 50–66 months). A flow chart of the study is shown in Figure 1.

### 2.2 Data collection

Demographic data were well collected and documented after training including gender, age, BMI, smoking, alcohol drinking history, and coexisting morbidities (hypertension, diabetes, hyperlipidemia, coronary artery disease [CAD], and stroke). CT or MRI was performed by qualified radiologists who are blind to the psychiatric status of patients within 72 h after admission. The National Institute of Health Stroke Scale (NIHSS) was applied to assess the severity of the stroke within 24 h of admission by trained neurologists, which provide reliable and valid information on stroke severity (Sun et al., 2006). According to the 17‐item Hamilton Depression Scale (HAMD) to assess depression status after admission, all subjects were classified as having either post‐stroke depression (PSD) (HAMD score ≥ 7) or non‐PSD (PSD) (HAMD score < 7) (Zhang et al., 2017).

### 2.3 Serum 25(OH)D

The serum concentration of 25(OH)D was measured using a competitive protein‐binding assay at the First Affiliated Hospital of Wenzhou Medical University's laboratory, within the first 24 h after admission. Serum 25(OH)D levels were recorded and divided into four quartiles (quartile 1: ≤38.4, quartile 2: 38.4– 54.6, quartile 3: 54.6−71.4, quartile 4: ≥71.4 nmol/L), as the raw vitamin D data were skewed.

### 2.4 Outcome assessment

The primary targeting outcome was assessed by modified Rankin scale (mRS) on follow‐up. The previous study has defined poor outcome as an mRS>2 (Pilz, Iodice, et al., 2011). Therefore, we defined the mRS as a dichotomous variable, an mRS score in the 0–2 range, which indicates no symptoms to a minimal disability, reflects good outcome, whereas the 3–6 range reflects moderate to severe disability or death, reflecting a poor outcome.

### 2.5 Statistical analysis

The relation of patient's baseline information and risk factors were statistically analyzed. The categorical variables were tested by chi‐square or Fisher's exact test and shown as percentage. Continuous variables have been expressed as mean ± SD and median (quartiles) depending on the normality of distribution and were compared by Student's *t*‐test or Mann–Whitney test, respectively. The risk factors for the development of poor outcome were analyzed by logistic regression analysis, adjusting for those conventional confounders and baseline variables related to poor outcome. Variables included in the multivariate logistic analysis were those found to be significant in our univariate analysis as well as those determined a priori based on clinical relevance. Model 1 included age, sex, body mass index, and baseline NIHSS score, whereas Model 2 involved the above 5 factors and medical history (diabetes mellitus, hyperlipidemia, hypertension, history of smoking, and alcohol drinking). Model 3 included the mentioned factors and stroke subtype in all stroke patients, and HAMD score at admission in AIS patients. IBM SPSS software was applied for analysis, and the results were expressed in odds ratio (OR) and 95% confidence interval (CI). *p* < .05 was considered statistically significant.

## 3 RESULTS

### 3.1 Baseline characteristics

After application of the eligibility criteria, 668 of 839 screened patients with stroke met the inclusion criteria and were enrolled in the study. After 5‐month follow‐up, 420 samples completed the follow‐up data: 270 of them were men (mean ± SD = 60.4 ± 10.7 years), while 150 were women (63.8 ± 9.0 years); note that 350 (83.3%) of the patients presented with AIS, while the remaining 70 (16.7%) showed ICH at admission; the median NIHSS score was 3 (1–5) and the mean 25(OH)D levels were 54.6 (38.3–71.5) nmol/L. There were no differences in baseline characteristics between those included in and excluded from the final analysis.

**FIGURE 2:**
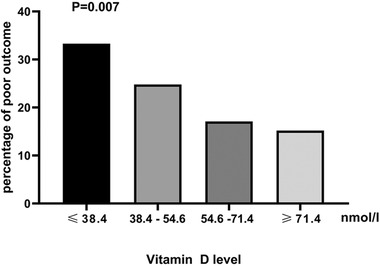
Percentage of patients in poor outcome group according to different vitamin D levels

Depending on the number of patients and the distribution of the 25(OH)D levels, the patients was divided into four layers to observe whether any enhanced performance could be quantified while maintaining statistical effect in each category: quartile 1: ≤ 38.4, quartile 2: 38.4–54.6, quartile 3: 54.6 –71.4, quartile 4: ≥71.4 nmol/L. An overview of baseline characteristics were concluded in gender, and alcohol drinking and history of diabetes differed by 25(OH)D quartiles levels (Table 1). Moreover, Figure 2 shows a significant intergroup difference in the incidence of poor outcome. Patients with vitamin D ≤ 38.4 were more likely to have poor outcome (33.3% versus 24.8%, 17.1%,15.1%, *p* = .007).

**TABLE 1:** Characteristics of stroke patients according to 25(OH)D levels

Group	Quartile 1 (≤38.4 nmol/L)	Quartile 2 (38.4–54.6 nmol/L)	Quartile 3 (54.6−71.4 nmol/L)	Quartile 4 (≥71.4 nmol/L)	*p*‐value
N	105	105	105	105	
Age (years), mean ± SD	59.66 ± 10.96	62.69 ± 10.34	61.36 ± 10.57	62.90 ± 8.71	.43
<65	65 (61.9%)	55 (52.4%)	61 (58.1%)	55 (52.4%)	
≥65	40 (38.1%)	50 (47.6%)	44 (41.9%)	50 (47.6%)	
Gender, male *n* (%)	61 (58.1%)	55 (52.4%)	71 (67.6%)	83 (79.0%)	.001
BMI (kg/m^2^), mean ± SD	24.04 ± 4.13	24.08 ± 3.23	24.00 ± 3.32	24.16 ± 3.20	.99
Education (years), mean ± SD	4.89 ± 4.23	3.91 ± 4.12	4.51 ± 3.83	3.14 ± 3.18	.008
Prior vascular risk factors					
Hypertension, *n* (%)	70 (67.3%)	84 (80.0%)	65 (63.7%)	71 (68.9%)	.06
Diabetes, *n* (%)	32 (30.8%)	33 (31.4%)	21 (20.8%)	14 (13.9%)	.008*
Hyperlipidemia, *n* (%)	12 (11.5%)	9 (8.6%)	7 (7.0%)	6 (5.9%)	.49
Coronary artery disease, *n* (%)	3 (2.9%)	8 (7.8%)	4 (4.0%)	6 (5.9%)	.40
Previous stroke, *n* (%)	16 (15.4%)	12 (11.4%)	5 (4.9%)	12 (11.9%)	.11
History of drinking, *n* (%)	27 (26.2%)	27 (26.2%)	29 (28.4%)	40 (39.6%)	.007*
History of smoking, *n* (%)	25 (26.3%)	34 (35.1%)	39 (41.9%)	43 (50.6%)	.11
Stroke subtype, *n* (%)					.007*
Intracerebral hemorrhage	17 (16.2%)	16 (15.2%)	18 (17.1%)	19 (18.3%)	
Acute ischemic stroke	88 (83.8%)	89 (84.8%)	87 (82.9%)	85 (81.7%)	
NIHSS, at admission, mean ± SD	3 (1 ± 6)	2 (1 ± 4)	3 (1 ± 4)	3 (1 ± 6)	.16
HAMD score, median (IQR)	6 (2–9)	3 (2–7)	4 (1.5–7)	5 (2–10)	.10
Poor outcome at 5 years, *n* (%)	35 (33.3%)	26 (24.8%)	18 (17.1%)	16 (15.2%)	.007*

Abbreviations: AIS, acute ischemic stroke; BMI, body mass index; ICH, Intracerebral hemorrhage; NIHSS, National Institutes of Health Stroke.

*p <0.005.

### 3.2 25(OH)D and 5‐years functional outcome

**FIGURE 3:**
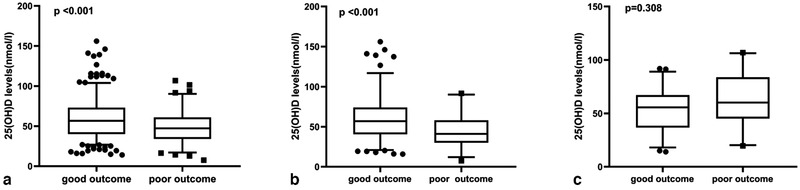
Comparisons of 25(OH) D between good and poor outcome: (a) in all stroke patients, (b) in AIS patients, and (c) in ICH patients. In the box‐and‐whisker plots, the horizontal line in the middle of each box indicates the median value; the lower and upper ends of the box represent the 25th and 75th percentiles

After 5‐years follow‐up, a total of 95 (22.6%) participants experienced poor outcome, while the total number of participants experienced the good outcome (mRS ≤ 2) was 325 (77.4%). Serum 25(OH)D levels (56.7 (40.1–73.2)) in patients experienced good outcome were significantly higher than those with poor outcome [(47.3(34.1–61.0) nmol/L, *p* < .001]. While a Student's *t*‐test found no significant difference in serum 25(OH)D levels or functional results in ICH patients (55.3 ± 22.2 vs. 62.7 ± 24.5, *p* = .31) (Figure 3).

Univariate analysis showed that higher age and admission NIHSS score, presenting with diabetes, hypertension, hyperlipidemia, and the history of smoking, were associated with poor outcome (Table 2).

**TABLE 2:** Clinical and demographic characteristics of all patients with good and poor outcome

Group	Good outcome	Poor outcome	*p*‐value
N	325	95	
25(OH)D (nmol/L), median (IQR)	56.7 (40.1–73.2)	47.3 (34.1–61.0)	<.001*
Age (years), median (IQR)	62.0 (55.0–68.0)	67.0 (58.0–72.0)	<.001*
<65	200 (61.5%)	36 (37.9%)	
≥65	125 (38.5%)	59 (62.1%)	
Gender, male, *n* (%)	212 (65.2%)	58 (61.1%)	.45
BMI (kg/m^2^), mean ± SD	24.2 ± 3.3	23.6 ± 3.8	.14
Education (years), mean ± SD	4.1 ± 3.9	4.1 ± 3.8	.93
Prior vascular risk factors			
Hypertension, *n* (%)	215 (67.2%)	75 (79.8%)	.019*
Diabetes, *n* (%)	69 (21.7%)	31 (33.3%)	.021*
Hyperlipidemia, *n* (%)	31 (9.8%)	3 (3.2%)	.044*
Coronary artery disease, *n* (%)	17 (5.4%)	4 (4.3%)	.68
Previous stroke, *n* (%)	31 (9.7%)	14 (14.9%)	.16
History of drinking, *n* (%)	114 (39.9%)	27 (32.1%)	.20
History of smoking, *n* (%)	106 (33.7%)	17 (18.1%)	.004*
Stroke subtypes, *n* (%)			.047*
Intracerebral hemorrhage	52 (14.9%)	24 (23.3%)	
Acute ischemic stroke	296 (85.1%)	79 (76.7%)	
Stroke recurrence, *n* (%)	13 (4.0%)	4 (8.2%)	.19
NIHSS at admission, mean ± SD	2 (1–4)	4 (2–6)	.001*
HAMD score, median (IQR)	4 (2–8)	6 (3–9)	<.001*
mRS at 5 years, median (IQR)	0 (0–1)	6 (4–6)	<.001*

Abbreviations: AIS, acute ischemic stroke; BMI, body mass index; HAMD, Hamilton depression scale; ICH, intracerebral hemorrhage; mRS, modified Rankin scale; NIHSS, National Institutes of Health Stroke.

*p <0.005.

Table 3 concluded that a multivariate‐adjusted logistic regression model was constructed where 5 years post‐stroke poor outcome as the dependent variable and 25(OH)D level over 71.4 nmol/L was considered as reference. After adjusting the confounders including age, gender, body mass index, medical history (diabetes mellitus, hyperlipidemia, hypertension, smoking, alcohol drinking), baseline NIHSS scores, stroke subtype, and HAMD score at admission, 25(OH)D ≤ 38.4 nmol/L remained significantly independently associated with the poor outcome (model 1: OR = 3.97, 95% CI = 1.78–8.84, *p *= .001; model 2: OR = 2.98, 95% CI = 1.22–7.25, *p* = .016; model 3:OR = 3.66, 95% CI = 1.42–9.45, *p* = .007). Moreover, younger age, lower initial NIHSS score, and the history of hypertension, diabetes, and smoking were also associated with poor outcome in multivariable analysis. An increasing risk of poor outcome was also observed among patients with ICH compared with AIS (OR = 4.06, 95% CI = 1.86–8.87, *p* < .001). As compared with patients aged <65 years, patients aged over 65 years also showed significantly increasing risk of poor outcome (OR = 4.25, 95% CI = 2.24–8.06, *p* < .001).

**TABLE 3:** Multivariate adjusted odds ratios for the association between serum levels of 25 (OH) D and poor outcome in all stroke patients

	Model 1		Model 2		Model 3	
25(OH)D (nmol/L)						
Q1	3.97 (1.78–8.84)	0.001*	2.98 (1.22–7.25)	0.016*	3.66 (1.42–9.45)	0.007*
Q2	2.27 (0.99–5.21)	0.05	1.44 (0.59–3.55)	0.42	1.75 (0.68–4.50)	0.25
Q3	1.40 (0.59–3.33)	0.44	0.98 (0.38–2.54)	0.97	1.14 (0.42–3.11)	0.79
Q4	1 (Reference)		1 (Reference)		1 (Reference)	
Age						
<65	Reference		Reference		Reference	
≥e5	4.04 (2.31–7.06)	<0.001*	3.75 (2.01–6.97)	<0.001*	4.25 (2.24–8.06)	<0.001*
NIHSS	1.12 (1.04–1.23)	0.006	1.15 (1.05–1.27)	0.003	1.17 (1.06–1.29)	0.002*
Stroke subtype						
AIS					Reference	
ICH					4.06 (1.86–8.87)	<0.001*

Abbreviations: AIS, acute ischemic stroke; CI, confidence interval; ICH, Intracerebral hemorrhage; NIHSS, National Institutes of Health Stroke; OR, odds radio.

Model 1: Adjusted for age, sex, body mass index, and baseline NIHSS score.

Model 2: Adjusted for covariates from Model 1 and further adjusted for medical history (diabetes mellitus, hyperlipidemia, hypertension, history of smoking, and alcohol drinking).

Model 3: Adjusted for covariates from Model 2 and further adjusted for stroke subtype.

Serum 25(OH) D levels in Q1 (**≤**38.4 nmol/L), Q2 (38.4−54.6 nmol/L),Q3 (54.6‐71.4 nmol/L), and Q4 (≥71.4 nmol/L).

*p <0.005.

**FIGURE 4:**
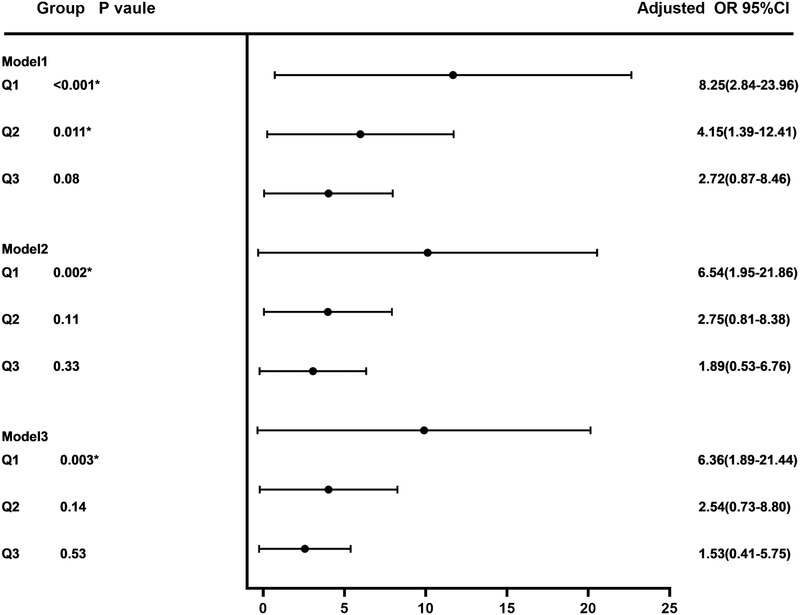
Multiple logistic regression between the vitamin D and functional outcomes at 5‐year post‐stroke OR: Odds radio, CI: Confidence interval, AIS: Acute ischemic stroke, NIHSS: National Institutes of Health Stroke. Model 1: Adjusted for age, sex, body mass index, and baseline NIHSS score. Model 2: Adjusted for covariates from Model 1 and further adjusted for medical history (diabetes mellitus, hyperlipidemia, hypertension, history of smoking, and alcohol drinking). Model 3: Adjusted for covariates from Model 2 and further adjusted for HAMD score at admission. Serum 25(OH) D levels in Q1 (≤38.4 nmol/L), Q2 (38.4–54.6 nmol/L),Q3 (54.6–71.4 nmol/L), and Q4 (≥71.4 nmol/L)

The effects of vitamin D levels on ischemic and hemorrhagic stroke were separately examined (Table 4). The result shows that vitamin D levels (≤38.4 nmol/L) were independently associated with the development of poor outcome in AIS (model 1: OR = 8.25, 95% CI = 2.84–23.96, *p* < .001; model 2: OR = 6.54, 95% CI = 1.95–21.86, *p* = .002). After adjusting for HAMD score at admission, 25(OH)D remained an independent outcome predictor, with an adjusted OR of 6.36 (95% CI = 1.89–21.44, *p* = .003; Figure 4). Vitamin D levels were not significantly associated with poor outcome of 5 years in ICH patients.

**TABLE 4:** Multivariate adjusted odds ratios for the association between serum levels of 25 (OH) D and poor outcome in AIS patients

	Model 1		Model 2		Model 3	
25(OH)D (nmol/L)						
Q1	8.25 (2.84–23.96)	<0.001*	6.54 (1.95–21.86)	0.002*	6.36 (1.89–21.44)	0.003*
Q2	4.15 (1.39–12.41)	0.011	2.75 (0.81–8.38)	0.11	2.54 (0.73–8.80)	0.14
Q3	2.72 (0.87–8.46)	0.08	1.89 (0.53–6.76)	0.33	1.53 (0.41–5.75)	0.53
Q4	1 (Reference)		1 (Reference)		1 (Reference)	
Age						
<65	Reference		Reference		Reference	
≥e5	3.51 (1.86–6.62)	<0.001*	2.89 (1.39–6.03)	0.005*	2.89 (1.32–6.33)	0.008
NIHSS			1.21 (1.08–1.36)	0.001*	1.21 (1.07–1.37)	0.003
HAMD score					0.99 (0.90–1.08)	0.78

Abbreviations: AIS, acute ischemic stroke, CI, confidence interval; OR, odds radio; NIHSS, National Institutes of Health Stroke.

Model 1: Adjusted for age, sex, body mass index, and baseline NIHSS score.

Model 2: Adjusted for covariates from Model 1 and further adjusted for medical history (diabetes mellitus, hyperlipidemia, hypertension, history of smoking, and alcohol drinking).

Model 3: Adjusted for covariates from Model 2 and further adjusted for HAMD score at admission.

Serum 25(OH) D levels in Q1 (**≤**38.4 nmol/L), Q2 (38.4−54.6 nmol/L),Q3 (54.6–71.4 nmol/L), and Q4 (≥71.4 nmol/L).

*p <0.005.

## 4 DISCUSSION

To date, it has been recognized that there were few large‐scale research to examine vitamin D levels and functional outcome of stroke patients with 5‐year follow‐up. In the present prospective observational study, we found 22.7% of acute stroke patients had poor outcome, and decreased 25(OH)D levels at baseline were independently associated with increased risk of poor outcome at 5 years after stroke in AIS, but not the ICH patients. This association was weakened after adjusting for HAMD score at admission, thus the effect of vitamin D levels has on functional outcome may be partially mediated by altering depression symptoms.

There were several population‐based reports on the relationship between vitamin D deficiency and cardiovascular disease, obesity, and dementia, and the higher risk of death as a result (Barnard & Colón‐Emeric, 2010). Previous studies also reported that vitamin D is an important biomarker for the poor outcome of stroke (Qiu et al., 2017; Tu et al., 2014; Wajda et al., 2019; Wei & Kuang, 2018). Daubail indicated that patients with 25(OH)D < 25.7 nmol/L have poorer functional prognosis compared with those stroke patients with 25(OH)D ≥ 25.7 nmol/L at discharge (Daubail et al., 2013). We suggested that vitamin D < 20 ng/ml contributed to 3.2‐fold increased risk of poor functional outcome in nondiabetic patients with AIS at 1 year after admission (Wei & Kuang, 2018). Daniela stated the vitamin D deficiency has a close connection with the outcome of AIS patients at 3‐month follow‐up. However, the studies mentioned are all based on a relatively short‐term follow‐up after stroke.

Here, we conducted a prospective study to investigate the relationship between vitamin D levels and the outcome of patients with 5‐year stroke including AIS and ICH. Standardized protocols and stringent quality control procedure were applied throughout the experiments including baseline data collection and outcome evaluation during the follow‐up. In addition, comprehensive information on potential confounders was collected and controlled in the multivariate model. After controlling these potential confounders, we found that vitamin D ≤ 38.4 nmol/L was significantly associated with an increased risk of poor outcome. Moreover, ICH patients were more likely to have a higher risk of poor outcome compared to AIS patients. Multiple mechanisms are mentioned upon the relation of low vitamin D levels and poor functional outcome after AIS. In the first place, inflammation responses have been strongly suggested to have a role in the pathophysiology of stroke. After the onset of stroke, the body presents a fast stress response, releasing a large number of inflammatory cytokines (Vila et al., 2003). Vitamin D produces a series of reactions referring to the anti‐inflammatory reaction by Treg and Th2, cytokine production, and pro‐inflammatory cytokine suppression, for instance, interleukin‐1, interleukin‐6, tumor necrosis factor‐alpha, and Toll‐like receptors. On the other hand, Vitamin D has been shown to reduce ischemia‐induced brain damage and suppress dysregulation of the inflammatory response after ischemia (Di Rosa et al., 2012; Wang et al., 2000). Consistently, in vivo studies also revealed that vitamin D deficiency exacerbates the proinflammatory state and suppressed neuroprotectants such as insulin‐like growth factor‐I (IGF‐I) (Balden et al., 2012).

Second, the main function of vitamin D is to regulate bone metabolism and maintain calcium‐phosphate homeostasis. Deficiency of vitamin D contributes to altered bone mineralization and low bone mass and accelerates bone resorption and reduction of bone density in patients with stroke (Lips & van Schoor, 2011; Pilz, Tomaschitz, et al., 2011; Yoshida & Stern, 2012).

Third, in vivo studies revealed that vitamin D3 supplementation decreased the volume of cerebral infarct, suggesting that vitamin D could exert neuroprotective effects (Milionis et al., 2014; Wang et al., 2000). Meanwhile, Vitamin D also upregulates the neurotrophic factors, such as nerve growth factor, neurotrophin‐3, neurotrophin‐4, and IGF‐I (Bogazzi et al., 2011). IGF‐I plays a significant role in post‐stroke recovery through regulating the repair of the central nervous system by promoting the germination and regeneration of axons and dendrites after stroke, and supporting the growth and survival of nerve progenitors, astrocytes, and microglial cells (Martino et al., 2011). It is also reported that IGF‐I may modulate the immune environment of the ischemic brain (Balden et al., 2012).

Additionally, Vitamin D deficiency increases the risk of cardiovascular disease by activating the renin–angiotensin system, leading to hypertension (Ginde et al., 2009; Lee et al., 2008). Vitamin D is also linked to diabetes, insulin resistance, and high BMI, which are risk factors for cardiovascular disease and all‐cause mortality. On the other hand, Vitamin D deficiency could give rise to poorer cognitive function and dementia, which affect the executive function and daily life of patients (Chen et al., 2018; Goodwill & Szoeke, 2017; Slinin et al., 2010).

Previous research have shown that lower vitamin D levels are associated with depression and AIS (Berridge, 2017; Hoogendijk et al., 2009; Larsson et al., 2018). Our team's previous study also have reported that vitamin D is an important biomarker for the occurrence of PSD (Han et al., 2015). In this study, after adjusting for HAMD score at admission, the effect of vitamin D levels on functional outcome was weakened. Therefore, the risk of poor outcome may be influenced by the interaction between vitamin D levels and depressive state.

However, in a latest double‐blind placebo‐controlled trial, vitamin D3 supplementation did not improve the recovery after acute stroke (Momosaki et al., 2019). There are no robust clinical trials regarding vitamin D supplementation to alter stroke outcome. Therefore, the underlying mechanism of the effect imposed by 25(OH) D on poor outcome may involve several convoluted pathological pathways, which remain to be elucidated.

There are still limitations in this study. The data were collected from a single study center and the sample size is still limited; a larger sample size would provide better sensitivity for the detection between the vitamin D levels and outcome. In addition, patients enrolled in the study were mild in most (median of NIHSS score was 3); the present findings could not be extrapolated to all AIS patients, and future research should add sample size to simultaneously assess the impact of vitamin D on different severity of stroke. Third, because the data were from past 5 years and most of the patients were mild, the number of patients involved in rehabilitation was small. Therefore, we could not evaluate the impact of rehabilitation in the observed associations. We did not have data on living habits such as exercise and diet, which may confound our conclusions.

## 5 CONCLUSION

In conclusion, this study has demonstrated that lower vitamin D levels are of long‐term prognostic significances after AIS within a 5‐year follow‐up. Vitamin D lower than 38.4 nmol/L is an independent prognostic marker for poor outcome after adjusting for possible confounding factors. Further analyses are anticipated to investigate the pathophysiological mechanisms and the role of vitamin D supplementation in the long‐term prognosis of stroke patients.

## CONFLICT OF INTEREST

The authors declare no conflict of interest.

## AUTHOR CONTRIBUTIONS

Ya‐Ying Zeng and Jin‐Cai He designed the study. Ya‐Ying Zeng and Cheng‐Xiang Yuan wrote the manuscript. Ya‐Ying Zeng did the statistical analyses. Meng‐Xuan Wu, Lin Cheng, Sheng‐Nan Zhou, Kai‐Li Fan, and Ping‐lang Hu screened and extracted data. Jin‐Cai He and Wen‐Jie Tang supervised study. All authors have made an intellectual contribution to the manuscript and approved the submission.

### PEER REVIEW

The peer review history for this article is available at https://publons.com/publon/10.1002/brb3.2244

## Data Availability

The data that support the findings of this study are available on request from the corresponding author. The data are not publicly available due to privacy or ethical restrictions.
